# Vertical interventions and system effects; have we learned anything from past experiences?

**DOI:** 10.11604/pamj.2015.21.262.6522

**Published:** 2015-08-07

**Authors:** Charlotte Oliveira, Giuliano Russo

**Affiliations:** 1 Instituto de Higiene e Medicina Tropical, Universidade Nova de Lisboa, Portugal; 2 Global Health and Tropical Medicine, Instituto de Higiene e Medicina Tropical, Universidade Nova de Lisboa, Portugal

**Keywords:** Ebola Outbreak Response, vertical interventions, global health initiatives

## Abstract

The recent Ebola Virus Outbreak had a devastating effect on West Africa's already feeble national health systems. We suggest that such an impact turned out to be catastrophic because it hit particularly hard human resources for health and the delivery of primary healthcare services, which are cross-sectional to any health system. National and international interventions failed to understand the nature of this interaction, and concentrated on attending urgent specific vertical functions to fight the outbreak - the pillars - such as surveillance, logistics, safe burials etc. Such patchwork and vertical intervention strategy was always going to fail to tackle a system-wide problem, particularly in already fragile systems. We suggest that future interventions will have to learn from the experience of past initiatives for the introduction of HIV-AIDS services, which started as vertical programs and ended up including ever growing health system strengthening components.

## Commentary

The devastating Ebola Virus Outbreak started hitting Western African Countries approximately one year ago; the first cases were reported on March 2014 in Guinea, and rapidly the epidemic spread into neighbor countries - Liberia, Sierra Leone, Nigeria and Senegal. After one year of widespread transmission of Ebola Virus Disease (EVD) nearly 24000 cases - confirmed and suspect - were reported, with over 9800 deaths [1]. After just five months the biggest EVD outbreak ever seen had brought to their knees already weak health systems, and - although belatedly - the WHO declared the current outbreak a Public Health Emergency of International Concern. A heterogeneous and at times fragmented international response then begun to unfold, and a plethora of international and national NGO's started responding to the outbreak without a clear coordination. International humanitarian response to this crisis has been described unceremoniously as late and uncoordinated by the very supposed coordinators [2]. The EVD outbreak hit directly as well as indirectly primary health care services in all the countries. In Sierra Leone, where the authors of this paper had the chance to operate, the majority of regular health centers rapidly stood idle, not only because of the reduced number of healthcare workers, but also because of the unexpected decrease of patients. We found that communities started avoiding the health facilities because of fear of being identified as suspect cases and of the stigma of being hold in quarantine for all the family and community. To compound such situation, we found EVD had an indirect effect on public health services such as on the reproductive health services of these countries, where quality services and skilled human resources were scaled down due to fear to deal with suspected Ebola cases in pregnant women [3]. We found that the international community unwillingly contributed to exacerbate the human resources for health shortage in ebola-affected countries; over the last year International and national NGO's started advertising ebola-related openings, offering different experiences and motivations to engage with the epidemic response - as well as sometimes higher salaries - to attract local staff already willing to trade their ill-paid jobs at Ministry of Health (MoH) departments for an NGO experience. This inevitably created a gap of human resources available to run regular National Health Service programs, particularly in primary care. In the Democratic Republic of Congo (DRC) focal points from different programs were appointed by the national MoH to integrate the NGOs task force during the Ebola outbreak; the unintended consequence of such a call was the interruption of regular activities in the Malnutrition and Tuberculosis programs, and well as in regular primary care visits. In Sierra Leone because of the simultaneous activities intended to curtail the trend of the epidemic, several Primary Health Units (PHU) were found often idle once all their regular health staff had been involved directly or indirectly in these activities, and regular services such as antenatal care and vaccination were interrupted for few days. In the EVD outbreak in DRC, to our surprise we found that at some stage regular patients started going to Ebola treatment centers to seek for primary care instead of the regular primary health centers, as they soon realized that the PHU couldn't respond to their basic needs, due to lack of medicines and medical equipment, while Ebola Treatment Centers (ETCs) were conspicuously well stocked. In a preexisting situation of poor service provision, ETCs were perceived by the population as an unexpected access door to decent health care, and this contributed to the reduction of demand for traditional primary health care services.

The creation of holding centers in Freetown, Sierra Leone, was merely conceived as a ‘band aid’ strategy to contain the epidemic, due to shortages of ETCs beds. Broadly speaking, these centers were only meant as a physical barrier where suspected cases could wait for the ambulance, or in severe cases, -the place where infected body could be isolated, until the burial team arrived. Even though these centers were not designed for treatment or service delivery, their health staffs were charged with managing the communication as well as coordination with central department to activate the system. As such staffs were also in charge for regular PHUs management and for other health activities, their new responsibilities created a functions overload, which exacerbated absenteeism and at time, interruption of regular health services such as assisted deliveries, antenatal care, under 5 consultation, and immunization campaigns. Interestingly a striking parallel could be drawn with the same constrains described in Mali and others countries following the scaling up of those Global Health Initiatives actions specifically focused on HIV-AIDS in those countries where disruption of basic health services was already the norm rather than the exception [4]. We have come to realize that a complex emergency such as the recent EVD outbreak, with over a year of devastating consequences, will always have a systemic impact among health systems, on its human resources, as well as on its supplies. However, reviewing the responses from different NGO's and MoH, we assisted to a fully-owned vertical strategy focus on the pillars-surveillance, safe burials, social mobilization, case management, logistics, communication, child protection and coordination-which was inherently inadequate to counteract EVD's system-wide shock. For example, a confirmed case of Ebola will always need interventions from all the pillars, from the moment that this person is admitted in an Ebola Treatment Center until the discharge; however, as the number of players involved in dealing with the case was high and the communication and coordination low, a gap in the response to the patient and community needs was soon created. Although the perverse effect of adopting vertical strategies to deal with system-wide health issues has been well-documented, particularly in the case of HIV-AIDS services [5], also for the present epidemic international NGO's resorted to design their intervention focusing on vertical strategies and ignoring the systemic impact of an outbreak. We suggest that the current EVD outbreak would have needed an integrated and systemic response. These affected countries health system's already have feeble sectors from (*workforce capacity to laboratory and other medical infrastructure, as well as a lack of adequate surveillance, information, and rapid response systems*.) [6]. The international response should have been more coordinated, strategic and proactive [7], learning from the recent mistakes done in a not so distant past. We believe that the response to the current EVD outbreak has not been different from responses to other epidemics and lessons learnt from the past - particularly when AIDS services were introduced in already feeble health systems - should have been taken in greater consideration, as decontextualized focus on HIV/Aids controlled to (*multiple and parallel coordinating bodies with a lack of leadership and overview*), as well as to a distortion of existent national policies and parallel monitoring and evaluation systems [5]. Also when high-cost AIDS services were first introduced by international NGOs in run-down facilities in the 2000s, patients rapidly learned how to play that unexpected provision of services to their advantage [8], and forcing international planners to include a system-strengthening component to their AIDS programs that could at least reduce the gap that was being created between AIDS-specific and regular services [9].

We believe that outbreak responses should be aimed at strengthening health systems, instead of piling on vertical and emergency activities. The strategy should be integrated in a comprehensive response from MoH and all stakeholders involved. A holistic EVD response focus on the pillars, but integrated on the comprehensive national health policies, could minimize the apparently inevitable gaps in health services delivery. In addition, a strong health workforce is a prerequisite for effective systems. Considering the impact of the current outbreak in human resources for health, the EVD outbreak response should have taken a systemic approach to such a scarce and volatile resource, promoting specialized training but also a generalized scaling-up exercise. The international response has been clearly delayed and uncoordinated. The impact of this fragmented response has led to parallel activities, overlapping with the fragile attempts of activities by the Ministry of Health. The focus on Ebola case management contributed to a weakening of the countries’ health structures and fragmented services. Health workforces were also affected, not only through EVD deaths among health workers, but also through the scaling up of daily activities by International actors. Lessons from past epidemics as well as from previous experience of introducing vertical services across pre-existing weak systems should have been reviewed and incorporated to the current response from all stakeholders. The possibility of an integrated and comprehensive response existed, and after years of outbreak emergencies and vertical strategies, the international aid response should have known better and focused on health system strengthening, even for such an apparently specific event.
